# Primary health care professionals’ experiences of using the Tilburg Frailty Indicator: an interview study

**DOI:** 10.1017/S1463423625100297

**Published:** 2025-07-18

**Authors:** Amelie Mazya, Anne-Marie Boström, Christina Sandlund, Anne Wissendorff Ekdahl

**Affiliations:** 1 Division of Clinical Geriatrics, Department of Neurobiology, Care Sciences and Society, Karolinska Institutet, Stockholm, Sweden; 2 Department of Geriatric Medicine of Danderyd Hospital, Danderyd, Sweden; 3 Theme Inflammation and Aging, Nursing Unit Aging, Karolinska University Hospital, Huddinge, Sweden; 4 Division of Nursing, Department of Neurobiology, Care Sciences and Society, Karolinska Institutet, Huddinge, Sweden; 5 R&D unit, Stockholms Sjukhem, Sweden; 6 Division of Family Medicine and Primary Care, Department NVS, Karolinska Institutet, Stockholm Sweden; 7 Academic Primary Health Care Centre, Region Stockholm, Stockholm, Sweden; 8 Department of Clinical Sciences Helsingborg, Lund University, Lund, Sweden

**Keywords:** Feasibility, frailty assessment, primary health care

## Abstract

**Aim:**

The aim of this study was to explore primary health care professionals’ (PHCP) experiences of frailty assessment with the Tilburg Frailty Indicator (TFI) with focus on feasibility aspects.

**Background:**

Primary health care (PHC) is often the first point of contact for older people and assessment of frailty is therefore often recommended in this setting. There is however a lack of awareness of frailty in PHC. The TFI has been proposed as a suitable instrument for frailty assessment in PHC. It consists of 25 questions, where ten questions aim to identify risk factors for frailty and 15 questions assess physical, psychological, and social frailty. There are no previous studies of feasibility aspects of TFI in PHC.

**Methods:**

A qualitative interview study with physicians, nurses, and physiotherapists that had used TFI in face-to-face interviews during a care visit. Interviews were transcribed and the text was thematically analyzed using qualitative content analysis.

**Findings:**

Nine interviews were performed. The PHCPs experiences were expressed in one theme: *TFI is useful and feasible but requires time and knowledge*. TFI was described as easy to use and providing a holistic assessment of the patient. Using the TFI was time-consuming but provided useful information for care planning. In conclusion, the TFI could be a clinically useful tool to assess frailty in PHC. The result indicates a need of educational efforts to increase knowledge about frailty and a need for primary health care to adjust to older people in order to allow care visits to include both assessment and management of frailty.

## Introduction

The upcoming changes in ageing demographics places increased demands on society and health care to address conditions that affect older people, such as multimorbidity and frailty. Frailty is regarded as a progressive, age-related decline in physiological systems which confers vulnerability to internal and external stressors and increases the risk of a range of negative health outcomes. (Cesari *et al.* 2016; World Health 2015) There are several proposed models of frailty where the biologically focused frailty phenotype by Fried et al and the accumulation of deficits theory by Rockwood et al. are dominating the literature. (Fried *et al.* 2001; Mitnitski *et al.* 2001) Gobbens et al has developed a third model of frailty: “frailty is a dynamic state affecting an individual who experiences losses in one or more domains of human functioning (physical, psychological, and social), which is caused by the influence of a range of variables and increases the risk of adverse outcomes.” (Gobbens *et al.*, 2010; Gobbens *et al.*, 2010a) The model emphasizes the importance of a holistic approach to the care of old people living with frailty, which also is recommended by the WHO. (World Health 2015) Moreover, a multidimensional approach to frailty is also more effective in both care planning and in preventive actions for frail older people. (Gilardi *et al.* 2018) The Tilburg Frailty Indicator (TFI) is based on this multidimensional and holistic model of frailty. (Gobbens *et al.*, 2010b)

The TFI questionnaire consists of 25 questions, where part A (ten questions) aims to identify risk factors for frailty and Part B (15 questions) assess physical, psychological, and social frailty. Only part B is included in the scoring, the established cut-off is five points or higher for frailty and the expected time it takes to complete is around 15 minutes. (Gobbens *et al.*, 2010b) TFI has a robust evidence of reliability and validity compared to other multicomponent frailty measures. (Sutton *et al.* 2016) A review by Gobbens et al of 27 studies recently summarized that results on internal consistency and test–retest reliability was noteworthy, as well as the criterion and construct validities. However, the association of the TFI with indicators of healthcare utilization, such as hospitalizations and visits to a general practitioner, was poor and studies concerning predictive ability had short follow-up periods. (Gobbens and Uchmanowicz 2021) There is also a need of further research of for example cross-cultural validity and responsiveness. (Zamora-Sánchez *et al.* 2022)

In 2013 the first international recommendation of frailty screening emerged after a frailty consensus meeting. (Morley *et al.* 2013) Later the International Conference on Frailty and Sarcopenia Research (ICFSR) recommended frailty screening using a simple, validated frailty instrument suitable to the specific setting or context. (Dent *et al.* 2019) The British Geriatric Society recommend assessment of frailty in any interaction between older people and health care or social professionals. (Turner and Clegg 2014)

Primary Health Care Professionals (PHCPs) are often first point of care contact for most older adults. Primary Health Care (PHC) is considered a suitable setting for frailty screening since most patients are not afflicted with acute illnesses or temporary convalescent. (Lacas and Rockwood 2012) Several factors need to be considered regarding assessment of frailty in the primary care setting. For example, besides choosing a valid and reliable instrument, time scale and need of resources are important. (Abbasi *et al.* 2018) There is also a lack of awareness of frailty in PHC in several countries. (Coker *et al.* 2019; Lacas and Rockwood 2012) A study showed that Italian PHCPs experienced difficulties in identifying both those who were frail and those who could become frail. (Obbia *et al.* 2020) Several studies concluded that there is a need of both training of the health care professionals (HCPs) and effective detection strategies in order to enhance assessment of frailty. (Coker *et al.* 2019; Kennedy *et al.* 2021; Obbia *et al.* 2020) In Sweden there are few studies of HCPs perceptions of frailty in older people. A qualitative study from 2011 including HCPs involved in providing care for older community-dwelling people, concluded that the HCPs views on frailty differed from the current knowledge about frailty. (Gustafsson *et al.* 2011)

TFI has been proposed as a suitable screening instrument for frailty in primary care and in public health. (Gilardi *et al.* 2018; Pialoux *et al.* 2012) A Swedish version of TFI has recently been validated and found to be adequately valid and reliable for frailty assessment in older, community-dwelling people. (Mazya *et al.* 2023) TFI has in previous research mostly been used as a self-administered instrument in a community setting. In order to collect information from older people not able to answer questionnaires by themselves, the TFI could be used by PHCPs during a care visit. There are however few, if any, studies regarding PHCPs’ experiences of using TFI as a questionnaire in face-to-face interviews, especially concerning feasibility. The aim of this study was to explore PHCPs’ experiences of frailty assessment with the TFI in a face-to-face administration, with focus on feasibility aspects.

## Methods

To increase the understanding of PHCPs experiences of frailty assessment with TFI, an exploratory qualitative study design was used, including individual interviews. Qualitative content analysis was conducted to develop categories and themes. (Graneheim *et al.* 2017; Graneheim and Lundman 2004; Lindgren *et al.* 2020) The COnsolidated criteria for REporting Qualitative research (COREQ) checklist was used as a quality reference for the report of this study. (Tong *et al.* 2007) The checklist is found in supplementary materials.

## Setting and participants

The study was conducted in PHC in Region Stockholm in Sweden. The municipalities where the participating PHCPs worked were situated both in the city and in the countryside and there were differences in socioeconomical circumstances between the municipalities. A purposive sampling approach was used. Registered nurses (RN), physicians (general practitioner residents, GPR) and physiotherapists (PT) were recruited from PHC-centres within the research and clinical network of the Academic Primary Health Care Centre (APHCC), a university health care unit of Region Stockholm’s PHC. Information about the study was given at online meetings organized by the APHCC and by e-mails to PHCPs. Those who were interested in participating in the study were recruited by the first author. PHCPs could be eligible for inclusion in the study if they had at least one year of work experience in PHC. The first author instructed the participants on how to use the TFI by oral and/or written information. Participants then selected two or three eligible patients that were 65 years or older and spoke Swedish. Patients with cognitive impairment, those who could not make an informed decision or those who were easily tired or afflicted by symptoms affecting their general condition, were not assessed with the TFI. The PHCPs assessed frailty with the TFI during a planned outpatient visit, after the patients had provided written consent.

### Frailty assessment in PHC

At the time of the study, assessment of frailty was not a part of clinical routine in PHC in Sweden. Some of the PHCPs were familiar with frailty assessment using the Clinical Frailty Scale (CFS). The TFI has to the authors knowledge only been used in a few Primary Care Centres, perhaps almost exclusively in research projects and has then been administered as a self-reported questionnaire.

## Data collection

A semi-structured interview guide was developed based on the research group’s professional knowledge on frailty, frailty assessment and experience in performing qualitative studies. The questions concerned the PHCPs’ experiences of using the TFI including feasibility, and their previous knowledge on frailty and assessment of frailty. The questions on feasibility were partly based on Bowen *et al*.’s feasibility framework (acceptability and practicality). (Bowen *et al.* 2009) The Swedish version of the TFI and the interview guide translated to English can be found in supplementary material. The first author conducted the interviews. Participants were encouraged to talk freely but probing and validating questions were used when needed. The PHCPs were interviewed via digital meetings and recorded by using a digital voice recorder. Only the interviewer and the participant were present during the interviews.

## Data analysis

The interviews were transcribed verbatim. Data analysis was performed using qualitative content analysis with an inductive approach. (Graneheim *et al.* 2017; Graneheim and Lundman 2004; Lindgren *et al.* 2020) The text was divided into meaning units and then condensed, abstracted and coded. The codes needed to be consistent with the context of the meaning unit and the whole text. The codes were compared regarding similarities and differences and sorted into sub-categories and then into categories based on the whole text and the authors’ impression of the underlying, not explicitly stated message. Finally, a theme was developed in order to interpret the result and demonstrate relationships between the experiences that addressed the research questions. An example of the analytical process is shown in table 1.

**Table 1.** Example of the analytical process

Meaning Unit	Condensed transcription	Code	Subcategory	Category
Interviewer: *Is there time to use this test (TFI) in your everyday clinical practice?* Respondent: *You could definitely use it during, for example, the admission process for home healthcare, since you have more time and no strict schedule to follow. I also think it could be used during an initial visit to your general practitioner, for instance, as you usually have more time for that. It wouldn’t be a bad idea to allocate some time for it in such cases.*	You could definitely use it during, for example, the admission process for home health care… an initial visit, perhaps in general, to your general practitioner, for instance	Useful for initial visits in home care and with general practitioners.	Can be used in multiple situations	Adaptable and adoptable

## Ethical considerations

The study follows the Declaration of Helsinki. (WorldMedicalAssociation 2013) Before data collection began, ethical approval was obtained from the Swedish Ethical Review Authority: Dnr 2022-01334-01. Participants, both PHCPs and patients, provided written informed consent before the interviews. Data was recorded and stored on a secure server provided by Karolinska Institutet.

## Reflexivity

The first author, a female specialist in geriatric medicine and at the time a PhD-student, had a pre-understanding of the subject of frailty and assessment of frailty. The first author was not working in PHC. The second author was a registered nurse with a postgraduate diploma in geriatric nursing and had experiences of working with frail older adults. The third author was a registered nurse with a postgraduate diploma in PHC nursing and had experiences of working in PHC. The last author was a specialist in geriatric medicine not working in PHC, with a wide experience in working with frail older adults and in geriatric research. The transcribed text was read repeatedly by the first and last author who separately structured the text into meaning units and codes and discussed the results together. Later the categories and possible themes where discussed within the whole author group, to ensure agreement on the interpretation of the text material. Quotations were used to illustrate the result.

## Results

**Figure 1.**
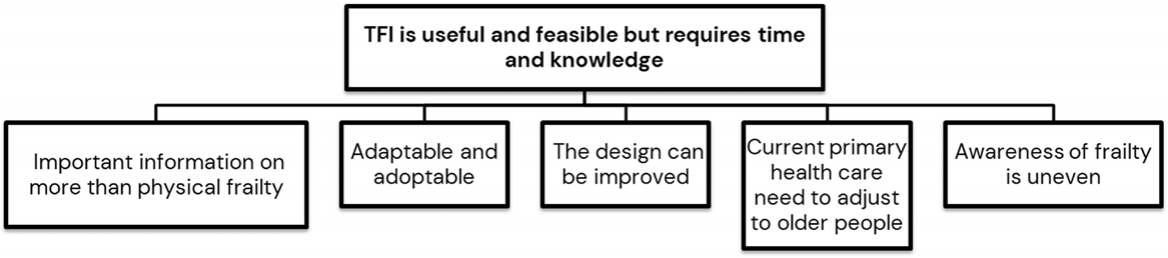
Overview of the theme and categories describing the PHCPs (primary health care professionals) experiences of frailty assessment with the TFI (Tilburg Frailty Indicator).

A total of nine participants between 29 and 54 years were recruited, of which seven were women and two were men. A detailed description of the participants is shown in table 2. The duration of the qualitative interviews with the PHCPs varied from 14 to 26 minutes, with a median of 21 minutes). The participants’ experiences of frailty assessment with the TFI was expressed in one theme built from five categories - TFI is useful and feasible but requires time and knowledge. (Figure 1)

**Table 2.** Characteristics of the primary health care professionals. Abbreviations: RN = Registered Nurse, GPR = General Practitioner Resident, PT = Physiotherapist, F = Female, M = Male, PHC = Primary Health Care, MNA = Mini Nutritional Assessment, CFS = Clinical Frailty Scale, ROAG = Revised Oral Assessment Guide, RUDAS = The Rowland Universal Dementia Assessment Scale, MMSE = Mini Mental State Examination, MoCA = The Montreal Cognitive Assessment, GDS = Geriatric Depression Scale, SMA = Safe Medication Assessment, PHASE 20 = PHArmacotherapeutical Symptom Evaluation 20

Participants	Profession	Sex	Work experience (PHC/total)	Experience of clinical measurement scales	Number of TFI-assessments
1	RN	F	15/15	MNA, Norton, CFS, Downton, ROAG	3
2	RN	F	12/12	RUDAS, MMSE, MoCA, MNA, CFS	2
3	GPR	F	3/7	GDS, CFS	3
4	RN	F	23/32	MNA, Downton, Senior alert, GDS, SMA, PHASE20, CFS	2
5	PT	F	14/14	Downton, Chair stand test, other measurements used by physiotherapists	2
6	RN	F	5/16	Downton, MNA, MMSE	2
7	GPR	M	6/6	CFS, Downton, Measurements for mental illness	4
8	PT	F	7/16	Fall prevention, Activity, and balance assessments	1
9	PT	M	7/9	Senior alert, Downton, other measurements used by physiotherapists	3

### Category 1. Important information on more than physical frailty

The PHCP’s overall appreciated the holistic approach to frailty by the TFI and found that much of the information from the assessment was beneficial both for medical decisions and a deeper understanding of the patients’ life and living circumstances. The PHCPs highlighted the questions on social frailty as important questions, especially if they had a long-lasting contact with a patient. An important consequence was that the PHCPs could evaluate if there was a need of more social support for the patients. “*It could, hands down, identify those who really needs to have people around them.” (PHCP 9)* The TFI was perceived as an instrument with questions covering dimensions of a patient’s health that otherwise could have been neglected. For example, the questions in part A were not usually asked and the PHCPs therefore expressed that indications of frailty previously not recognized became more evident after the assessment, which was an eye-opening experience.

The questions about psychological frailty and social frailty were described as questions that patients were not used to receive and that made them even more important to ask. “*Since it is questions that I normally do not ask, frailty - that I did not think about earlier – can appear.” (HCP 5)* Those with earlier experience of frailty assessment had used the CFS and described the information from the TFI as more comprehensive due to the inclusion of the social and psychological dimensions of frailty, which were perceived as relevant. It was hard to single out what was the most important information received from the assessment with TFI, the PHCPs concluded that the big picture was more important than information on separate questions. “*I think you should take this time and do a thorough assessment and see all parts of a person’s health and life, it cannot be that narrow that you only see your part*.” *(PHCP 5)*

The PHCPs found that assessment of frailty with TFI could be helpful in planning the future approach or set-up of the care, for example put the patient on a waiting list for at least yearly visits to the physician or RN, increase the frequency of RN visits or contact the municipality in order to assess the need of more social care. The question about if one feels physically healthy was perceived as important by the PHCPs. The patient’s description of him-/herself as frail, healthy, capable or other was a valuable perspective. “*Yes, it is important that even if you are well, it is highly relevant how you perceive your own health, I believe.” (PHCP 3)*

### Category 2. Adaptable and adoptable

Some PHCPs expressed that they would have preferred if the patients had filled in the TFI by themselves before the outpatient visit, others saw no problem in using it during the visit. The PHCPs saw a potential use in connection with a first care contact. Some of the PHCPs suggested alternative ways of using the TFI, for example in a digital format, as a template in the electronical record or as it was originally intended, as a self-administered instrument to be filled out for example in the waiting room. Some appreciated the instrument as a basis for discussion during the visit. “*Then you can see* (on a prefilled questionnaire) *and perhaps ask the patient, here you have answered that you have difficulties, what should we focus on today?” (PHCP 2)* The PHCPs overall found that the TFI could be useful when meeting a new patient. Other potential situations where the TFI could be useful were in connection to yearly visits, medication reviews and at enrolment in home-based care. “*But you could absolutely use it at admittance to home care, or overall at a first visit to your General Practitioner.” (PHCP 3)*

The PHCPs described the TFI as user-friendly and direct and the questions as succinct and simple, they did therefore not see a need for a specific education about the TFI before using it. It was proposed that the best way to learn more about the TFI could be to employ it in clinical practice. It was suggested that the competence gained during the basic training for each profession of the PHCPs should be enough in order to use the TFI. Others suggested that the need of education depended on previous experience in using clinical measurement scales.

### Category 3. The design can be improved

Sometimes only parts of the TFI were useful. PHCPs raised concern that patients perhaps might not understand or had difficulties in answering the question about if one’s lifestyle is healthy or not, and if one has difficulties in coping with problems. The question about chronic disorders also seemed to be problematic for some patients to understand, the PHCPs had to explain to the patient what the question was about and then together with the patient reach an answer. The PHCPs experienced that the question about if one felt physical healthy depended on how the patient perceived their physical health. The PHCPs perceived that there were patients that perhaps never had reflected on these questions and therefore had a hard time to answer. “*I think the hardest thing to find out actually is how they view their health.” (PHCP 8)* The PHCPs experienced that the patients often wanted to have a third answer alternative – sometimes - for the questions that only hade yes/no alternatives. PHCPs understood that the answers had to be yes or no in order to be able to calculate a score.

The question on income in part A, determinants of frailty, was described as uncomfortable and awkward to ask. The PHCPs were concerned that the patients would react negatively and be hesitant to answer, which was the case with one patient. Most patients had no specific reaction and one even told the PHCPs that it was a relevant question. Another reflection from the PHCPs was that the patients perhaps could have answered this question directly on the questionnaire before the assessment, in order to circumvent a potential uncomfortable situation. It was also suggested that the question could be rephrased to if “one could handle unexpected costs.” One PHCP reflected that some older people perhaps could be hesitant to reveal information about their economic status due to fear of fraud.

The scoring process was regarded as complicated by several of the PHCPs, partly due to the design of the TFI questionnaire with the scoring instructions on a separate page. Others found it easy to calculate the points. To increase user-friendliness, adding a separate scoring column at the same page as TFI part B was suggested. The instructions to the scoring were also perceived as complicated by some. The PHCPs described that they for some patients were surprised by the total score of the TFI, some of the patients that appeared robust according to the PHCPs clinical judgement scored high on the TFI. This was somewhat unexpected and caused some concern, the PHCPs reflected that patients with a positive mind set who were happy and enjoyed social interaction perhaps appeared less frail even though they scored high on the TFI. There were also patients with a low score and a robust appearance that did not feel well. “*Even if I was a bit surprised by the total points at the end, I did some reflection….and yes, indeed this patient was frail and even very frail.” (PHCP 7)* The PHCPs found that some questions of the TFI part A were redundant since they were usually asked during outpatient visits.

### Category 4. Current primary health care need to adjust to older people

The PHCPs concluded that there was a lack of awareness about frailty in PHC and that training in frailty assessment and discussions about frailty with other PHCPs would widen the clinical competence. “*But perhaps some kind of education about frailty would be good, because the knowledge is not so good, colleagues that I work with don’t really know what it (frailty) is and why you should assess it.” (PHCP 2)* The PHCPs described several circumstances that would make them use the TFI for frailty assessment. One situation that would facilitate employment of the TFI was if it was incorporated as a template in the electronical medical record. It was ascertained that their employer needed to implement mandatory clinical routines for frailty assessment before they would start using it. “*If it comes from above that this is what you should do, you make sure to do it.” (PHCP 1)* Another motivation for frailty assessment was that frailty was regarded as better than age to identify those in need of care.

Almost all the PHCPs commented on the TFI as time-consuming when used as a face-to-face interview. The patients gave long, detailed answers on several of the questions, that consumed the whole visit. PHCPs also needed to take time to explain some questions and even if this could lead to further discussions about health and care, the time it took was overall perceived as too long.

PHCPs also described that they would have been happy to do frailty assessments more often if they had scheduled time for it, meaning that the current health care system did not allow visits long enough to include a frailty assessment. “*There are many clinical measurements and scales that you could use more, but it is the time!” (PHCP 8)* The PHCPs raised concerns and reflected on how to handle the results of the frailty assessment with TFI. Some were concerned about the question about exposure to crimes and that they did not know how to handle if someone answered yes on that question. There was a suggestion that a written text with suitable actions or interventions next to the questions of the TFI could help the PHCP to take the next step. “*So if I start asking these questions, then it is me that need to follow-up and not let it go.” (PHCP 1)*

### Category 5. Awareness of frailty is uneven

The PHCPs’ experience in frailty assessment varied. There were few who in their daily work assessed frailty with or without an instrument. The CFS was the only frailty assessment instrument that previously had been used. The PHCPs described that they assessed conditions related to frailty, such as risk of falls, risk of pressure ulcers, risk of malnutrition and overall health assessments. They also explained that they through knowledge of patient history and assessment of function could get an impression if the patient was frail or not. The PHCPs believed there was an intuitive assessment of frailty when they tried to get an overall picture of the patient and that frailty as a concept articulated that intuitive judgement. “*This person will soon fall apart.” (PHCP 6)* When the PHCPs were asked about what characterizes a frail person, answers were diverse and influenced by uncertainty. The PHCPs described a condition often constituted by multimorbidity and psychological and social impairments, foremost loneliness. Others added malnutrition and low weight as important hallmarks of frailty. Impaired function and need of assistance in daily function was included as important aspects of frailty. Frailty was also described as a fluctuating state where a new medical condition could have great negative impact on both overall health and quality of life of the frail older person. “*It must be when the (patient’s) state varies much from day to day, they can be lonely and depressed….I don’t really know how to pinpoint it…” (PHCP 9).*

During the interviews, PHCPs were asked about how different grades of frailty could be described. It was challenging to answer the question and there was variation in the answers. Some depicted a frailer person as more afflicted with multimorbidity and functional impairment while others thought that a frailer person would have low weight and anxiety. The PHCPs mentioned cognitive impairment and number of hospitalizations as gradients for frailty. Many described that an increased need of help in daily activities was the hallmark of increased grade of frailty.

When the PHCPs were asked about their experience of interventions for frailty, several suggestions emerged. The PHCPs described a need of involving several health care professions in the care of the frail patient based on the current conditions that afflicted the patient. Decreasing loneliness, introduction of physical activities and nutritional support were interventions and/or preventive measures suggested by the PHCPs. Continuity of care, meaning recurrent visits to/from the same RN and physician, was also suggested as important in the care of frail older people. Actions aiming to increase social support could be involving relatives, contacting social services regarding social care in the home and initiating daytime activities offered by the municipality. “*To include the whole team around the patient in order to get a holistic assessment, so we don’t miss anything….talk to everyone to ensure that we can make the patient feel safe.” (PHCP 9)*

## Discussion

The aim of this study was to examine PHCPs’ experiences of assessment of frailty with the TFI with focus on feasibility aspects. To our knowledge this is the first study that explores PHCP’s experiences of frailty assessment using the TFI in face-to-face interviews during an outpatient visit. The main results from this study was formulated in one theme: TFI is useful and feasible but requires time and knowledge.

Our findings will be discussed in terms of acceptability and practicality, two aspects of Bowen et al.’s feasibility framework.(Bowen *et al.* 2009) Acceptability can be described as to what extent a new measure is judged as suitable, satisfying or attractive by the users. Practicality refers to what extent the measure can be carried out with intended participants using existing means, resources and circumstances.

Our results indicate that the overall acceptability of the TFI was high among the PHCPs. They described the TFI in positive terms and were appreciative of the holistic picture of the patients’ health that the TFI provided. Several PHCPs expressed that the TFI provided important and suitable questions, especially those with information on social frailty. Although the PHCPs regarded the question on income as important, several felt uncomfortable asking the patients about their income. This was not foreseen in the ethical discussion during the planning of the study. Reactions to disclosing financial difficulties could be different depending on cultural context, financial information for example is considered private in Sweden, hence asking about money could feel intrusive. Financial struggle could also lead to feelings of shame and therefore difficult to discuss, although this could depend on cultural context. How the patients in this study experienced the question about income cannot be answered, although the PHCPs conferred that only one patient was hesitant to answer, and another patient expressed that is was an important question. Low socioeconomic status is a well-known risk factor for frailty. (Dugravot *et al.* 2020) Routine collection of socioeconomical data is therefore important in health care. In a recent study, patients in Primary Care were comfortable answering question on income, if they understood the connection between income and health, and believed the data would be used to improve care. (Pinto *et al.* 2022) In a qualitative study regarding older peoples understanding of frailty, participants mentioned limited financial capacity as an important aspect of frailty. (Golbach *et al.* 2024) Hence, asking about economy in a health care setting might not be a tense situation after all. Instead of asking about monthly income a question on financial strain could be a more suitable way of asking about economy or – Do you have money for unforeseen expenditure? This was also suggested in the previous mentioned study regarding asking for information on income in PHC. (Pinto *et al.* 2022)

PHCPs were concerned that the instruction to calculate the score was difficult. They did however only assess two or three patients, so perhaps only more training would facilitate the scoring. There was also a suggestion that the design could be improved to ease the scoring. It was suggested that a more suitable way of applying the TFI was to only use part B, since part A confers information often available to the PHCPs. This would shorten the frailty assessment and therefore make it more feasible to perform which connects to the practicality of the TFI. The acceptability of the TFI could be improved if some of the proposed changes were performed. On the other hand does part A (risk factors for frailty) convey important information for HCPs contributing to the holistic picture of the patient. Awareness of the assessed risk factors could lead to a more individualized care management.

The TFI was originally designed as a self-reported questionnaire to be used in public health but in this study, we interviewed PHCPs who had used TFI in face-to-face interviews. The PHCPs expressed diverse opinions regarding the best mode of using the TFI. PHCPs appreciated that the face-to-face mode provided a base for a structured conversation during the visit and gave opportunity for explanation and discussion of questions that was hard to answer, thus deepening the understanding of the patient’s health status. Potential situations when the face-to-face mode seemed more advantageous and acceptable than the self-report mode were: meeting a new patient, yearly visits, medication review-visits, and at enrolment in home-based care. On the other hand, a pre-filled questionnaire would decrease the time for the visit, perhaps making it more practical and feasible, at the potential loss of more detailed information. However, a previous study of frailty screening in community-dwelling older people found that self-administered instruments had lower completion rates compared to nurse-administered instruments, indicating that face-to-face administration might be more successful for frailty screening in Primary Health Care. (Ambagtsheer *et al.* 2020)

The participants’ experiences regarding practicality of the TFI highlights the lack of resources, foremost time, in order to enable frailty assessment. Lack of time is an eloquent example of how health care today is not adjusted to old people. Another limitation was the lack of guidelines for assessment and management of frailty in PHC. This connects to the low awareness and understanding of frailty in PHC as reported in earlier studies. (Coker *et al.* 2019; Kennedy *et al.* 2021; Lacas and Rockwood 2012; Obbia *et al.* 2020) In our study the PHCPs expressed diverse and uncertain descriptions of frail older people, which is consistent with earlier findings and implicates a need for education. Several aspects of practicality need to be improved in order to increase the feasibility of frailty assessment with TFI or other frailty instruments.

The gold standard of care for older people with frailty is by involving a multi-professional team with geriatric competence - Comprehensive Geriatric Assessment (CGA). (Clegg *et al.* 2013; Kim and Rockwood 2024) CGA-based interventions include an assessment of medical, psychological, functional, and social aspects of the older patient. CGA-based care in a geriatric outpatient setting have effect on mortality and can delay progression of frailty. (Ekdahl *et al.* 2016; Mazya *et al.* 2019) In this study the PTs were employed by rehabilitation centres and the GPRs and RNs were employed in primary care centres. Both the PHCPs in primary care and the PTs from rehabilitation expressed that they could contact one and another, and even social services in the municipality, if they saw the patient needed this competence. This suggest that even if not employed as a team in the same organization, PHCPs can involve other care professions around a patient and thus create the prerequisites for a team-based care, with or without a political or organizational support. This is beneficial for frail older patients.

## Strengths and limitations

Confirmability was ascertained by following the steps of analysis according to Graneheim and Lundman, and repeated discussions between the authors. (Graneheim and Lundman 2004) Credibility was ascertained by including participants with different professions, thus providing various perspectives. The credibility may have been lowered due to low number of participants. However little new information was received in the last interviews, which indicates that data saturation was reached. Adequacy of sample size can be assessed in different ways in qualitative studies. The concept of *information power* introduced by Malterud et al. indicates that the more information the sample holds, relevant for the actual study, the lower number of participants is needed. Considering the study’s narrow aim, purposive sampling and the use of an interview guide to increase clarity in the communication, the information power could be regarded as rather high, meaning a need of fewer participants. (Malterud *et al.* 2016) Participants who volunteered were however similar in age, mostly female and interested in frailty and/or the care of older people, somewhat limiting the variability. The risk of inconsistency of data was minimized in this study since an interview guide was used. This approach might however have limited the dialogue. The interviewing skills developed during the data collection which could have led to richer material in the later interviews. In order to strengthen the transferability of this study, the participants, the setting and the process of data analysis were described in detail.

Since frailty assessment will become increasingly important, we believe that our findings can provide knowledge valuable for future health care. (Kim and Rockwood 2024) Future studies need to include additional key persons such as occupational therapists, psychologists, dieticians, assistant nurses and administrative personnel to broaden the perspectives on feasibility and clinical utility of the TFI. Also, the question regarding the best way of administration need to be addressed. Explorative studies of older patients’ experiences of TFI is also crucial before changes to TFI or implementation of frailty assessment with TFI could be considered at a larger scale.

## Conclusion

Results from this study emphasize frailty as an elusive but important concept in care management of older people. If frailty assessment with the TFI should be feasible in clinical routine, PHCPs need education, guidelines about care (prevention and treatment) of older living with frailty, support and more allocated time for outpatient visits of older patients from the funding bodies.

## Clinical implications

Identification and management of frailty will be increasingly important in health care, especially in PHC and there is a need of valid and reliable frailty assessment instruments that also are feasible to perform. The WHO recommend a holistic approach to the care of older people. The TFI is a holistic frailty assessment that could be used as a self-administered questionnaire or face-to-face by PHCPs during an outpatient visit in order to facilitate care planning if PHCPs are provided with education and allocated time for assessment of older patients.

## Supporting information

Mazya et al. supplementary material 1 Mazya et al. supplementary material

Mazya et al. supplementary material 2 Mazya et al. supplementary material

Mazya et al. supplementary material 3 Mazya et al. supplementary material

## Data Availability

Data is available upon request by contacting the first author.

## Supplementary material

For supplementary material/s referred to in this article, please visit https://doi.org/10.1017/S1463423625100297
