# Supplementary material for: Primary health care professionals’ experiences of using the Tilburg Frailty Indicator: an interview study
Source: Prim Health Care Res Dev. 2025 Jul 18;26:e61. doi: 10.1017/S1463423625100297 (PMC12281041; doi:10.1017/S1463423625100297)
Supplement: Mazya et al. supplementary material 2 — Mazya et al. supplementary material [file S1463423625100297sup002.docx]

**INTERVIEW GUIDE – ASSESSMENT WITH TFI IN PRIMARY CARE**

**Introduction**

Inform about frailty and TFI, recap after previous oral and written information given before the TFI assessments.

Inform about the study, its purpose and refer to the written information sent via email.

Inform about the length of the interview (approx. 15 min, max 30 min), that the interview will be recorded, and the freedom to withdraw.

Inform about confidentiality, anonymization, and written consent.

Obtain written consent (photographed or scanned).

**Information about the interviewee**

| Occupation: |  |
| --- | --- |
| Gender: |  |
| Age: |  |
| Workplace: |  |
| Years in the profession: |  |
| Years in primary care: |  |
| Previous experience using assessment scales in patient work at the health centre? If yes, in which area? |  |

**RECORDER ON**

**----------------------------------------------------------------------------------------------------------**

**PURPOSE:** To investigate whether healthcare professionals have prior knowledge about frailty.

1. Can you describe what characterizes patients with frailty/different degrees of frailty?
2. Are you aware of any interventions that can prevent or treat frailty?
3. Do you currently assess the degree of frailty/risk of frailty? If yes, which assessment form?

**PURPOSE:** To investigate healthcare personnel’s experiences with frailty assessment using the Tilburg Frailty Indicator.

4. What did you think of the TFI form?

a. Overall

b. Part A (Background factors)

c. Part B (frailty assessment)

d. Response options? For some questions, you could answer Yes, Sometimes, No.

5. Did the patient express any opinions about the questions in the TFI?

a. Content

b. Wording

6. Do you have any opinions on: (feasibility factors)?

a. Time required

b. Material required

c. Patient acceptance

d. Interviewer’s acceptance

e. Is training needed to perform the test?

f. Is there room to use the test in clinical practice?

7. Would you consider using TFI for frailty assessment in the future?

a. Comments?

8. Does information about frailty add anything to

a. Your assessment of a patient?

b. Your decision on further investigation?

c. Your decision on treatment?

d. Which information is most essential?

9. Do you take any actions if you assess a patient as frail, regardless of whether you used an assessment scale?

10. Is the information obtained from the frailty assessment with TFI worth the time it took to complete the instrument?

11. Is there anything you would like to mention that you think is relevant beyond what I have asked, or something you would like to elaborate on?

------------------------------------------------------------------------------------------------------------

**RECORDER OFF**

Thank the interviewee for their participation.

Provide the person with contact details of the responsible researcher for any questions.
