# Supplementary material for: Primary health care professionals’ experiences of using the Tilburg Frailty Indicator: an interview study
Source: Prim Health Care Res Dev. 2025 Jul 18;26:e61. doi: 10.1017/S1463423625100297 (PMC12281041; doi:10.1017/S1463423625100297)
Supplement: Mazya et al. supplementary material 3 — Mazya et al. supplementary material [file S1463423625100297sup003.pdf]

Tilburg Frailty Indicator (TFI)*

Gobbens RJJ, van Assen MALM, Luijkx KG, Wijnen-Sponselee MTh, Schols JMGA. The Tilburg Frailty Indicator: psychometric properties. J Am Med Dir Assoc 2010; 11(5):344-355.

Del A Bakgrundsfaktorer

1. Vilket kön tillhör du?	0 Man	0 Kvinna
2. Hur gammal är du?	.... år	
3. Vilket är ditt civilstånd?	0 gift/sambo<br>0 ogift<br>0 frånskild/har separerat<br>0 änka/änkeman	
4. I vilket land är du född?	0 Sverige<br>0 Om annat land, vilket?<br><br>…………………………………………	
5. Vilken utbildning har du? Ange den högsta.	0 Grundskola/Folkskola<br>0 Gymnasieutbildning<br>0 Universitets- eller högskoleutbildning	
6. Vilken kategori beskriver ditt hushålls månadsinkomst efter skatt?	0 Under 10 000 kr<br>0 10 001 – 15 000 kr<br>0 15 001 – 20 000 kr<br>0 20 001 – 30 000 kr<br>0 30 001 – 45 000 kr<br>0 45 001 kr eller mer<br>0 Vet inte	
7. Hur hälsosam tycker du att din livsstil är överlag?	0 Hälsosam<br>0 Varken hälsosam eller ohälsosam<br>0 Ohälsosam	
8. Har du två eller fler kroniska sjukdomar?	0 ja	0 nej
9. Har du upplevt någon av följande händelser under det senaste året?		
- en närståendes död	0 ja	0 nej
- en allvarlig sjukdom hos dig själv	0 ja	0 nej
- en allvarlig sjukdom hos en närstående	0 ja	0 nej
- en skilsmässa eller avslutat ett nära förhållande	0 ja	0 nej
- en trafikolycka	0 ja	0 nej
- varit utsatt för ett brott	0 ja	0 nej
10. Är du nöjd med din boendemiljö?	0 ja	0 nej

Del B Frågor om skörhet

B1 Fysiska aspekter

11. Känner du dig fysiskt frisk?	0 ja		0 nej
12. Har du senaste tiden ofrivilligt gått ner mycket i vikt? *("mycket" innebär 6 kg eller mer under de senaste sex månaderna, eller 3 kg eller mer under den senaste månaden)*	0 ja		0 nej

Upplever du problem i ditt dagliga liv på grund av:

13..........svårigheter att gå?	0 ja		0 nej
14.......... svårigheter att hålla balansen?	0 ja		0 nej
15………nedsatt hörsel?	0 ja		0 nej
16………nedsatt syn?	0 ja		0 nej
17. ....nedsatt styrka i händerna?	0 ja		0 nej
18. ....fysisk trötthet?	0 ja		0 nej

B2 Psykologiska aspekter

19. Har du problem med minnet?	0 ja	0 ibland	0 nej
20. Har du känt dig nedstämd den senaste månaden?	0 ja	0 ibland	0 nej
21. Har du känt dig nervös eller orolig den senaste månaden?	0 ja	0 ibland	0 nej
22. Klarar du av att hantera problem på ett bra sätt?	0 ja		0 nej

B3 Sociala aspekter

23. Bor du ensam?	0 ja		0 nej
24. Saknar du att ha människor omkring dig?	0 ja	0 ibland	0 nej
25. Får du tillräckligt mycket stöd från andra människor	0 ja		0 nej

* TFI är översatt till svenska från engelska enligt metoden för back-translation med tillstånd från instrumentkonstruktören RJJ Gobbens.

Poängsättning Del B Skörhet (Skala från 0 till 15)

Fråga 11:	ja = 0<br>nej = 1
Fråga 12-18:	ja = 1<br>nej = 0
Fråga 19:	ja = 1<br>nej och ibland = 0
Fråga 20 och 21:	ja och ibland = 1<br>nej = 0
Fråga 22:	ja = 0<br>nej = 1
Fråga 23:	ja = 1<br>nej = 0
Fråga 24:	ja och ibland = 1<br>nej = 0
Fråga 25:	ja = 0<br>nej = 1

Gräns för skörhet: ≥ 5

Amelie Lindh Mazya
amelie.lindh.mazya@ki.se
Specialistläkare Geriatrik
Doktorand Karolinska Institutet
Stockholm 2019
